# Authentic Versus Simulated Patient Videos: Effect on Mental Status Examination—An Educational Randomized Controlled Trial

**DOI:** 10.1007/s40596-024-02111-5

**Published:** 2025-01-22

**Authors:** Nicolaj Mikkelsen, Esben Blaabjerg Larsen, Sidse Marie Arnfred

**Affiliations:** 1 Psychiatric Research Unit, Mental Health Service Region Zealand, Slagelse, Denmark; 2 Copenhagen University, Copenhagen, Denmark

**Keywords:** Medical students, Psychiatry, Randomized controlled trial, Mental status examination, E-learning

## Abstract

**Objective:**

This study evaluated the impact of adding authentic patient video training to a base e-module featuring simulated patient videos, aimed at improving the mental status examination (MSE) skills of fifth-year medical students during their psychiatric rotation.

**Methods:**

A randomized controlled trial (RCT) was conducted with 290 students, assigned to either an experimental group, the full e-learning group (Full), or an active comparator group, the limited e-learning group (Limited). The Limited group received a base e-module on MSE, while the Full group received both the base e-module and an additional module with 23 authentic patient videos. MSE accuracy was assessed digitally 1 week after each rotation through multiple-choice tests based on unseen video cases. Self-reported engagement with the modules was also analyzed.

**Results:**

Of the 290 enrolled students, 190 completed at least one MSE test. No significant improvement in MSE accuracy was observed in the Full group compared to the Limited group (10.1 vs. 9.9; *F*_1,188_, 0.152; *p* = 0.7). Increased engagement with the videos, both within and across groups, did not lead to better MSE outcomes.

**Conclusion:**

The addition of authentic patient videos did not significantly enhance MSE skills compared to simulated videos within the digital-only format of the study. Engagement with the video library did not influence the outcomes. The study adds to the ongoing conversation about the efficacy of e-learning in medical education, emphasizing the need for caution when adopting e-learning approaches without integrating blended learning strategies.

The psychiatric interview—including the mental status examination (MSE)—has been a cornerstone of psychiatric practice since Karl Jaspers’ foundational work on psychopathology in 1913. The MSE entails a thorough evaluation of a patient's cognitive, emotional, and behavioral functions and is essential not only in psychiatry but also in broader medical practice; for instance, mental disorders are involved in an estimated 25% of family practice consultations [1, 2]. Proficiency in writing a MSE using psychiatric terminology is an essential skill for medical students to acquire during their clinical psychiatric rotation [3, 4].

The educational landscape of the psychiatric rotation is increasingly strained by challenges like expanding class sizes, shortened rotation periods, a shortage of psychiatrists, and fewer inpatient beds [5–8]. Consequently, clerkship students face fewer patient interactions and reduced bedside teaching, making it difficult to grasp complex concepts such as the MSE, which relies heavily on hands-on learning for effective skill development [9–12].

To address these challenges, video-based training has emerged as a promising educational tool [13–15]. Videos can break down complex procedures like the MSE into manageable segments, enabling close observation of signs and symptoms [16, 17]. Additionally, videos of both authentic and actor-simulated patient interviews, the latter also referred to as standardized patients, have been reported to boost student engagement and recall [5, 18–20]. An ethnographic study found that clerkship students during their psychiatric rotation benefited from authentic patient videos, using them to practice clinical assessments, mimic interview skills, and bridge the gap between theory and practice [21].

A scoping review on teaching strategies for the MSE highlights that video interviews tend to yield higher student satisfaction and better learning outcomes compared to lectures and reading material alone [1]. Digital learning tools are not only cost-effective but also provide consistent exposure to a diverse array of clinical presentations, irrespective of the clinical context of a clerkship rotation [5, 22, 23].

Driven by this rationale, an e-library of video vignettes featuring *authentic patient interviews* was developed as part of a preliminary study aimed at supplementing bedside MSE training for fifth-year medical students at Copenhagen University (CPHU) during their psychiatric rotation. The goal was to enhance students’ proficiency in conducting MSEs [3, 21, 24, 25]. The preliminary study suggests that students who utilized the video e-library during clinical rotations performed better on MSE tests compared to those in standard rotations without such resources [3], demonstrating its potential to improve MSE skills in clerkship settings with limited educational support.

Utilizing authentic patient videos, rather than simulated videos, was deemed relevant to accurately depict the diverse, real-world manifestations of psychiatric conditions [26]. Building on the promising results from the preliminary study, we performed a randomized controlled trial (RCT) to compare the impact of *authentic versus simulated* patient interview videos on MSE skills among clerkship students, thereby evaluating two variations of video-based training.

Writing an MSE from a psychiatric interview requires complex clinical reasoning [27]. Video recordings of these interviews simplify the learning process by allowing students to focus solely on observation, detached from the complexities of direct patient interaction and treatment planning [19]. Although this method lacks live patient engagement, it provides a safe learning environment where information can be broken down into manageable parts, consistent with medical education theory [28]. This structured approach can then be strengthened through subsequent direct patient encounters.

Along these lines, research in psychiatry and medical education shows that videos of authentic or simulated patients improve engagement and recall, and may aid in transferring skills to clinical practice [5, 19, 20]. One study shows that videos featuring simulated patient portrayals of psychopathology significantly enhance the teaching of MSE for a diverse group of preclinical medical students [23]. Further efficacy research supports this, demonstrating that self-directed video training results in higher test scores compared to traditional lectures, even those that include detailed MSE videos [29]. Additionally, exposure to authentic patient videos has shown advantages over lectures in terms of improving students’ ability to conduct MSEs, as evidenced by performance in multiple-choice tests [30, 31] and live interview scenarios [32].

To further investigate the educational benefits of authentic patient videos for MSE skills in psychiatric clerkship students, we conducted a fully digital RCT to build upon the findings from the preliminary study. Our goal was to determine whether authentic patient videos offer an *additional* educational effect over simulated patient videos in enhancing MSE skills, addressing the limited research comparing different digital teaching methods for the MSE, as no study has directly compared authentic and simulated videos in this specific context [1, 31]. Additionally, we aimed to control for potential confounding factors, such as variations in teaching quality across CPHU-affiliated psychiatric hospitals and units.

The purpose of this study was to investigate if additional access to an authentic patient video e-library improved MSE skills in clerkship students compared to those with access solely to simulated patient video content. We hypothesized the following: (1) students with access to both the authentic patient video e-library and the e-module with simulated patient videos would achieve higher MSE test scores compared to students with access only to the simulated patient video e-module. (2) There would be a positive correlation between time spend on the e-learning modules—within and across the groups—and the student’s MSE test scores.

## Methods

### Ethics and Data Security

This study was approved by the CPHU Ethics Committee for Health and Science and by the Danish Data Security Agency. Patients provided informed written consent to participate in the educational videos. Enrolled students signed a study consent form, and their data were kept on a secure server. Access to the video e-library was granted only after students acknowledged detailed confidentiality rules, which included restrictions on downloading, taking screenshots, or sharing content with unauthorized individuals. The video e-library administrator manually granted and revoked access for each student during their clinical attachment. An agreement for data transfer from Region Zealand to CPHU was signed to facilitate access to the video e-library on a CPHU server.

### Design

This study was a parallel group, superiority, partial-blinded, cluster-RCT. Both interventions were an addition to the regular 3-week psychiatric rotation at the fifth year of medical school at CPHU. The trial included two groups of clerkship students: the *experimental group*—full e-learning (Full)—which had add-on access to both the authentic patient video e-library and the e-module with simulated patient videos, and the *active comparator group*—limited e-learning (Limited)—which had access only to the e-module with simulated patient videos.

Clerkship students complete their psychiatric rotations at 11 different facilities across the Island of Zealand. We performed cluster randomization based on clerkship location, including only students who consented to participate. Cluster randomization was conducted according to hospital location. Blinding of students and local lecturers to the allocation groups was not possible. The unblinded primary investigator (SMA) collected the informed consent digitally through SurveyXact© (IBM), checked the names on the clerkship student enrollment list from the faculty, and performed cluster randomization at the clerkship location level. Students who consented received individual invitations to the designated e-module. The researcher (EBL) conducting the follow-up assessment—a digital MSE skill test—was blinded to intervention allocation. Statistical analysis was also performed by a researcher blinded to group allocation, with masking only being lifted after the primary outcomes had been analyzed and conclusions formulated. The study protocol was registered and published on ClinicalTrials.gov (ID: NCT05795387) on March 20, 2023, prior to analysis of the primary outcome.

### Participants

Fifth-year medical students allocated to the clinical psychiatric rotation from CPHU during three consecutive semesters in 2022 and 2023 were included in the study, provided they gave written consent. Students who had previous access to the e-library were excluded. In a preliminary study [3], nearly all eligible participants enrolled, but a 26% drop-out rate was observed by the time of the final MSE test. Based on the observed differences in MSE test scores—1.9 points between medical doctors and students, and 0.9 points between different student groups—a meaningful difference was set at 1.25 test points. Assuming a standard deviation between 1.6 and 2.4, we set it at 2.0 for this calculation. To detect this difference with adequate statistical power, we estimated a required sample size of 88 students per group, resulting in a total of 176. Allowing for potential drop-out, the target enrollment was set at 220 students.

### Study Context and Procedure

In Denmark, the medical program consists of a three-year bachelor’s degree followed by a 3-year master’s degree with a clinical focus. At CPHU, students spend the first three terms of the clinical master’s program in internal medicine and surgery through lectures and clerkships. By the fourth term, when the clinical psychiatry rotation occurs, students are already familiar with hospital settings and clinical documentation.

CPHU assigns fifth-year medical students to the mandatory clinical psychiatry rotation. Each term, approximately 240 students are allocated across 11 different psychiatric hospitals or units. After completing a 1-week lecture series on psychiatry, the students participate in a mandatory 3-week psychiatric rotation at one of these designated facilities. During this period, students shadow physicians in inpatient wards, gaining practical experience in conducting psychiatric assessments, documenting patient cases, proposing diagnoses, and formulating initial treatment plans. The semester culminates with an oral long case examination, assessing their proficiency in clinical psychiatry [3].

At the beginning of the term, the research team sent study information via emails to all fifth-year medical students via the learning management system. One week before the initial lecture week, an additional email was sent to a shift of 60–70 students, requesting their consent to participate. During the lectures, the study details were presented verbally, and students provided electronic consent using SurveyXact. Consenting students were entered into the data management system and randomized by the end of the first lecture week. On the first day of clinical psychiatric rotation, participating students were informed about their group allocation and their eligibility for accessing one of the two e-learning modules at their university learning management platform.

Participating students underwent the MSE skills test via Zoom-webinars following the clinical rotation. Questionnaires were administered via SurveyXact at the beginning and end of the clinical rotation. As part of the recruitment strategy, students who participated in the study and completed the MSE skills test were granted access to the e-library with authentic patient videos prior to the end-of-term oral long case exam in clinical psychiatry. However, the exam itself was not part of the current study. The study was active for three semesters in 2022 and 2023.

### The Intervention: MSE E-learning Modules

The e-library with authentic patient videos contained 23 video vignettes of brief patient interviews with adjoining MSEs written by three faculty psychiatrists [3, 24]. Patients for the authentic videos were recruited from a general psychiatric hospital comprising four integrated wards, an emergency unit, and several outpatient clinics. Interviews began with questions about recent or present mental issues, followed by screening for affective disorders, psychosis symptoms, and self-harm risk. Recordings lasted 8–20 min, with final video vignettes edited to 8–12 min, as recommended [20]. Some videos included a psychiatrist explaining various elements of the MSE. Diagnoses were omitted to maintain focus on the descriptive task. Following instructional design recommendations for video teaching [17, 19, 33], we provided written instructions for students to read a brief note about the patient, watch the video, write their own MSE, and compare it with the provided expert MSE summary.

The Full group had access to the authentic patient video e-library and another e-module with simulated patient videos. The latter included written textbook-style explanations, a glossary, a cartoon, and eight videos with an actor simulating patients, all regarding MSEs. The Limited group only had access to the e-module with simulated patient videos plus the shared written material on MSE. This ensured that any differences in scores could be attributed to the addition of authentic patient videos.

### Measures

The baseline questionnaire collected data on age, gender, clinical rotation unit, interest in psychiatry, and prior clinical psychiatric experience. Other questionnaires were also administered at baseline, with findings to be reported elsewhere. The *primary outcome* was the clerkship students score on the MSE skill test administered the week following clinical rotation. The test comprised three subtests, each featuring a video vignette not available in the library, ensuring that none of the students have seen it before. Following each video, students completed a forced choice multiple-choice questionnaire (MCQ) using SurveyXact [3]. The MSE skill test was carried out on Zoom ©.

After watching each video, students had 5 min to complete the forced choice task opened in SurveyXact ©. Each MCQ contained 20 descriptive one-sentence statements that could be used in an MSE. Students had to mark the five statements they believed most accurately described the case. Of these 20 statements, five were correct, five were detrimentally wrong, and ten were not to the point. A custom scoring algorithm was applied: correct statements, + 3 points; detrimentally wrong statements, − 3 points; not to the point statements, 0 points. The score for each test ranged from − 15 to + 15 points. The overall MSE skills test score was the average score of the three subtests.

*Secondary outcomes* included student feedback on the e-learning features, focusing on student engagement. Additionally, analyses were conducted on the MSE test scores for individual video cases to determine variation across different patient presentations. Data on time spent on the e-learning modules was collected by student self-reporting and by primitive digital tracking data from the learning management system. For the Full group, further analyses included the self-reported number of videos watched, aiming to explore potential correlations between engagement and MSE test performance.

### Statistical Analysis

Data was handled and analyzed in SPSS v.29 ©. Descriptive data was presented as means and standard deviations, frequencies, and percentages. Data condensation was employed, reducing five category responses to fewer meaningful categories. Distribution differences was analyzed in chi-square tests. The primary outcome was analyzed using an intention-to-treat (ITT) approach without imputation, as no information could inform it. We applied analysis of variance (ANOVA), comparing the two allocation groups, and post hoc a two-factor ANOVA including allocation group and sex. Pearson correlations were applied to investigate associations between the primary outcome and previous experiences with clinical psychiatry, interest in psychiatry, and self-declared time spend on the learning modules time. Student feedback scores was analyzed in simple ANOVAs.

## Results

Out of 584 clerkship students invited, 290 enrolled and were randomized. Among them, 190 students (65.5%) participated in a MSE skills test: 98 in the Full group and 92 in the Limited group. A total of 100 students (51 from the Full group and 49 from the Limited group) did not attend any testing sessions. The dropout rate was 34.2% for the Full group and 34.8% for the Limited group.

Table 1 outlines that the two groups were generally well-matched in terms of age (Full group 27 years, SD 2.9; Limited group 26 years, SD 2.8), sex, interest in psychiatry, and consideration of becoming a psychiatrist. Most participants reported “none or sporadic” clinical psychiatric experience (90.8% in the Limited group and 83% in the Full group). Conversely, 17% of the Full group reported “some or more” experience, compared to 9.2% in the Limited group. The difference in clinical psychiatric experience was statistically significant (chi-square 3.810, df = 1; *p* = 0.05).

**Table 1:** Baseline characteristics of study participants. This table presents the demographic and clinical characteristics of participants in the full e-learning (Full) and limited e-learning (Limited) groups. *Chi-square 3.810; df = 1; *p* = 0.05

		Limited group		Full group		Total	
		*N*	%	*N*	%	*N*	%
Sex	Male	38	27.0%	55	36.9%	93	32.1%
	Female	103	73.0%	94	63.1%	197	67.9%
	Total	141	100.0%	149	100.0%	290	100.0%
Clinical psychiatric experience*	None or sporadic	128	90.8%	122	83.0%	250	86.8%
	Some or more	13	9.2%	25	17.0%	38	13.2%
	Total	141	100.0%	147	100.0%	288	100.0%
Psychiatric interest	Not interested	41	29.1%	41	27.9%	82	28.5%
	Indifferent/do not know	56	3.7%	54	36.7%	110	38.2%
	Interested	44	31.2%	52	35.4%	96	33.3%
	Total	141	100.0%	147	100.0%	288	100.0%
Consideration of becoming a psychiatrist	Not interested	71	50.4%	71	48.3%	142	49.3%
	Indifferent/do not know	52	36.9%	55	37.4%	107	37.2%
	Interested	18	12.8%	21	14.3%	39	13.5%
	Total	141	100.0%	147	100.0%	288	100.0%

As shown in Table 2, our primary outcome—the mean MSE test score—for the Full group was 10.1 (SD 2.6), while the Limited group scored 9.9 (SD 2.9). This difference was not significant (*F*_1,188_, 0.152; *p* = 0.7). Moreover, there was no group difference observed in scores on subtest videos (p-range 0.3–0.8). Notably, female students demonstrated higher MSE accuracy compared to male students across intervention groups (*F*_1,188_, 6.294; *p* = 0.01), and particularly for one subtest video a marked sex difference was observed in the Limited group (*F*_1,88_, 6.765; *p* = 0.01).

**Table 2:** Mental Status Examination Test Scores. This table summarizes the primary outcome, comparing the MSE test scores between the full e-learning (Full) and limited e-learning (Limited) groups using an intention-to-treat approach. Significant sex differences, mean values in bold mark p > 0.01. *F1,189: 6.294, *p* = 0.01; **F1,184: 8.030, *p* = 0.005; #F1,89: 6.765, *p* = 0.01

		Limited group			Full group			*Total sample*		
		*N*	Mean	STD	*N*	Mean	STD	*N*	Mean	STD
Video 1	Male	*25*	**7.6**	4.7	*31*	9.1	2.9	*56*	**8.4**	3.9
	Female	*65*	**10.1**^**#**^	4.0	*64*	10.3	4.1	*129*	10.2**	4.0
	Total	*90*	9.4	4.3	*95*	9.9	3.8	*185*	9.7	4.0
Video 2	Male	*25*	8.8	5.0	*31*	8.6	4.4	*56*	8.7	4.6
	Female	*64*	9.3	4.8	*65*	9.2	4.6	*129*	9.2	4.7
	Total	*89*	9.1	4.8	*96*	9.0	4.5	*185*	9.1	4.6
Video 3	Male	*25*	10.7	3.9	*32*	11.4	3.8	*57*	11.1	3.8
	Female	*67*	11.5	2.7	*65*	11.9	2.5	*132*	11.7	2.6
	Total	*92*	11.3	3.1	*97*	11.8	3.0	*189*	11.5	3.0
MSE Test	Male	*25*	9.0	3.3	*32*	9.5	2.9	*57*	**9.3**	3.1
	Female	*67*	10.3	2.8	*66*	10.4	2.4	*133*	10.3*	2.6
	Total	*92*	9.9	2.9	*98*	10.1	2.6	*190*	10.0	2.8

Student feedback on e-learning features is summarized in Table 3. The key finding is that the Full group reported significantly more hours spent on the MSE e-modules compared to the Limited group. The mean time spent by the Limited group was 3.3 h (SD 2.1), while the Full group spent 5.1 h (SD 3.9) (*p* = 0.001).

**Table 3:** Student feedback. This table summarizes student feedback on the time spent engaging with the e-learning modules in the full e-learning (Full) and limited e-learning (Limited) groups. ^#^Likert scale 1–5, where 5 is most positive. *F 11,748; df 151.1; *p* = 0.001

	Limited group			Full group			Total		
	N	Mean	*STD*	N	Mean	*STD*	N	Mean	*STD*
Limited and Full group									
Overall, how satisfied have you been with the clinical rotation in psychiatry?	90	3.6	*1.1*	97	3.5	*1.1*	187	3.5	*1.1*
How many MSEs have you completed during your clinical rotation?	90	4.8	*2.9*	97	5.0	*2.7*	187	4.9	*2.8*
How many hours have you spent in total on the project's MSE e-modules?*	69	**3.3**	*2.1*	84	**5.1**	*3.9*	153	4.3	*3.4*
To what extent do you find the learning platform satisfactory as an e-learning tool?	67	4.1	*0.9*	83	4.3	*0.9*	150	4.2	*0.9*
Full group only									
Approximately how many authentic patient videos have you watched?				56	7.3	*5.2*			
To what extent did the authentic patient videos help you learn how to conduct an MSE?				56	4.4	*0.7*			
How effective were the audio explanations for the authentic patient videos?				45	3.6	*0.9*			
How effective was the use of multiple-choice questions in relation to the videos?				47	4.3	*0.7*			

Mean values in bold mark *p* < 0.001

There was no relationship between the time spent on the modules and test performance, neither within nor across groups (total sample* r* − 0.004, *p* = 0.9, *N* = 135), nor was there any association with interest in psychiatry (total sample* r* − 0.064, *p* = 0.4, *N* = 190). We found no significant correlation between the number of authentic patient videos watched and MSE scores for the students in the Full group (total sample* r* − 0.077, *p* = 0.6, *N* = 49).

## Discussion

In summary, this study aimed to evaluate whether an add-on authentic patient video e-library could enhance MSE skills among fifth-year medical students in psychiatric rotation beyond the effect of an add-on e-module with simulated patient videos. The findings—contrary to our hypothesis—suggest that access to authentic patient videos did not significantly improve MSE accuracy scores, nor did increased engagement with the videos, both within and across groups, translate into better MSE outcomes.

We found that the Full group did not significantly outperform the Limited group in identifying correct MSE statements. This lack of efficacy may be partly due to when and how the e-modules were introduced. It is possible that the cognitive load of the clerkship limited full engagement with the additional e-learning, and some students may have delayed viewing the material until just before exams, thereby reducing their educational value during the intended study period. The timing and context, therefore, could be potential confounders masking the true effect of the authentic videos on MSE skills.

Analysis on individual videos showed no significant differences between groups across the three scenarios used in follow-up assessments. This consistency suggests that add-on authentic patient videos did not enhance specific MSE skills targeted by diverse patient cases.

Table 1 reveals a notable difference in psychiatric clinical experience between the two groups*,* with 17% of the Full group reporting “some or more” experience compared to 9.2% in the Limited group. Although this added clinical experience could theoretically enhance the application of knowledge from the authentic video modules—a concept supported by a small study showing that experienced learners outperform novices in MSE identification in an online test with simulated videos [2]—it did not translate into improved test scores in our study.

Despite the Full group reporting over 50% more time spent with the e-modules, they did not achieve better MSE test scores compared to the Limited group. This highlights a crucial aspect of educational interventions: Increased engagement with learning materials, as expanded on later, does not necessarily translate into improved learning outcomes. The same tendency is observed within the Full group itself. In the analysis of the Full group only, neither the number of authentic videos viewed nor the time spent on the modules showed a significant correlation with MSE accuracy. This contrasts with a preliminary study that demonstrated notable improvement in students who watched more than seven videos [3].

A noteworthy finding, albeit secondary to the focus of this study, was the significant difference in performance based on sex. Female students exhibited greater accuracy in selecting correct answers, regardless of their group assignment; see Table 2. The preliminary study [3] yielded similar unpublished results, and we speculate if it indicates inherent differences in how male and female students observes mental states. This observation warrants further investigation.

The literature emphasizes that video-based clinical training is valued for its repeatability and clinical realism, often enhancing MSE teaching compared to traditional methods [20, 29–31, 34]. However, there is limited research comparing different digital teaching methods for MSE training [1]*.* Our study aimed to address this gap and found that access to authentic patient videos did not improve MSE skills beyond simulated videos, nor did an increase in engagement correlate with better MSE performance.

A study in the broader field of medical education, where evidence on video modality effectiveness likewise remains limited [35], examined first-year medical residents using authentic patient-consultations for video-based feedback to enhance self-awareness and communication skills. Despite reported improvements in self-awareness, no significant differences were observed in empathy or communication skills between groups using authentic versus simulated videos only [36]. This aligns with our findings, suggesting that the authenticity of patient videos alone does not guarantee better educational outcomes, and perceived effectiveness does not necessarily translate to improved objective scores.

In a preliminary study [3], the use of authentic video-based learning, supplemented with instructor-led sessions, significantly improved MSE skills compared to a control group with no video resources. This *blended learning* approach combined video content with face-to-face instruction, allowing instructors to contextualize the material. In contrast, the *fully digital* format used in this RCT, which lacked instructor interaction, did not yield similar improvements in MSE skills despite increased engagement from the Full group. Moreover, in the preliminary study, all e-learning occurred on-site during the clinical rotation, fostering opportunities for peer discussion and teamwork. A qualitative analysis revealed that students frequently watched the videos together, discussing their relevance to clinical practice [21]. In the current study, however, students had the option to view the videos from home, with only 11% engaging in peer viewing (data not otherwise reported). This may indicate that the educational benefits of authentic videos for clerkship students can plateau in the absence of structured instructor support and on-site peer interaction.

Supporting this notion, research on self-regulated online learning emphasizes the importance of instructional guidance, highlighting that engagement alone—without instructor feedback or peer interaction—may not enhance learning outcomes [37]. Therefore, the way videos are integrated into the learning process, particularly through blended approaches, appears vital for maximizing their educational impact.

A 2016 systematic review and meta-analysis [38] assessed studies comparing blended learning with non-blended methods among health professional learners. The analysis found that blended learning had a large positive effect (SMD 0.81, 95% CI 0.57–1.05) compared to non-blended learning, including both traditional face-to-face instruction and *pure e-learning*. This suggests that blended learning, which incorporates interactive elements like exercises, peer discussions, and instructor feedback, is more effective for improving knowledge retention and application. Similarly, a 2019 systematic review echoed these findings, demonstrating that blended digital education, the combination of digital and traditional methods, holds significant potential for enhancing communication skills and knowledge acquisition among medical students [39].

To build on these findings and enhance the understanding of video-based learning for MSE training, future research should explore blended learning approaches that combine e-learning with in-person tutoring and discussion at clerkship sites. If further studies reveal that authentic and simulated videos offer comparable educational benefits, simulated videos should be favored due to their advantages in safeguarding patient privacy.

Several limitations for the study must be considered. The dropout rate in both groups was relatively high but within expected norms. A review of 71 RCTs in leading medical journals found that 18% of trials reported dropout rates of 20% or higher [40]. Furthermore, the dropout rate was similar across both groups, suggesting it was unrelated to the type of intervention or participant characteristics, likely reflecting the demanding schedule of clerkship students.

A key limitation was the inability to blind students and local medical professionals to group allocation, which may have introduced performance or detection bias. Students in the Full group, aware of their enhanced resources, might have felt compelled to perform better, while those in the Limited group may have been less motivated. Similarly, lecturers and medical professionals aware of group assignments might have unintentionally altered their interactions with students, creating an uneven learning environment. However, it is important to note that the follow-up assessment and statistical analysis were conducted by researchers blinded to group allocation. Despite these concerns, no significant differences in MSE scores were found between the two groups suggesting that bias of these categories likely did not play a major role in the final outcomes.

A strength of our study is that our primary outcome was based on an objective measure rather than relying solely on student evaluations, which is common in medical education. However, some measures did rely on self-reported data, which is inherently susceptible to bias. This applies to both student feedback across groups and the number of authentic videos watched by students in the Full group. While recall errors could have influenced reporting, we find no clear incentive for systematic under- or over-reporting. However, it is possible that students in the Full group, aware of their study status, may have exaggerated their engagement with the video modules.

Another limitation in this study is the uncertainty regarding the sensitivity and specificity of the MSE skills test. Because the test has not been fully validated, it is possible that it was not sensitive enough to detect subtle improvements in MSE skills developed from watching authentic patient videos. These videos focus on real-world variability in psychiatric symptoms, which may not have been fully captured by the test’s format or scoring algorithm. However, it is worth noting that in the preliminary study, a reference group of clinicians—including 30 psychiatrists and 27 psychiatric residents—scored higher on the MSE test than both student groups [3], providing some degree of validation for the test.

Lastly, the brief exposure period to authentic patient videos (3 weeks) may have been insufficient for students to fully develop their MSE skills. Although a longer follow-up might yield different results, it would undermine the study’s goal of assessing the impact of these videos within the practical constraints and limited resources typical of a psychiatric clinical rotation.

In conclusion, the add-on authentic video-based training module did not significantly enhance the MSE skills of fifth-year medical students beyond the base e-module. Engagement with the video library did not influence the outcomes. The study contributes to the ongoing dialogue about e-learning as an effective educational strategy for medical education, cautioning the implementation of purely digital approaches without blended elements.

## Data Availability

Data is available by contacting the last author, Sidse Marie Arnfred, per email: sidar@regionsjaelland.dk.
